# The Impact of Digital Storytelling on Presence, Immersion, Enjoyment, and Continued Usage Intention in VR-Based Museum Exhibitions

**DOI:** 10.3390/s25092914

**Published:** 2025-05-05

**Authors:** Sungbok Chang, Jungho Suh

**Affiliations:** 1 Department of Digital Media Design, College of Arts, Cheongju University, Cheongju 28496, Republic of Korea; sbchang@cju.ac.kr; 2 Department of Media Communication, College of Social Science, Gachon University, Seongnam 13120, Republic of Korea

**Keywords:** VR exhibition, digital storytelling, presence, immersion, enjoyment, reuse intention

## Abstract

Recent advancements in virtual reality (VR) technology have introduced a new paradigm in exhibition culture, with digital storytelling emerging as a crucial component supporting this transformation. Particularly in virtual exhibitions, digital storytelling serves as a key medium for enhancing user experience and maximizing immersion, thereby fostering continuous usage intention. However, systematic research on the structural influence of VR-based digital storytelling on user experience remains insufficient. To address this research gap, this study examines the impact of key components of digital storytelling in VR—namely, interest, emotion, and educational value—on presence, immersion, enjoyment, and continuous usage intention through path analysis. The results indicate that interest, emotion, and educational value all have a significant positive effect on presence. Furthermore, while interest and emotion positively influence immersion, educational value does not show a statistically significant effect. Presence, in turn, has a positive effect on immersion, enjoyment, and continuous usage intention, while immersion also positively influences enjoyment and continuous usage intention. Finally, enjoyment was found to have a significant positive effect on continuous usage intention. This study empirically validates the effectiveness of digital storytelling in virtual exhibition environments, offering valuable academic and practical insights. Theoretically, it contributes to the field by elucidating the complex and hierarchical relationships among three core factors—interest, emotion, and educational value—and their impact on user experience. Practically, the findings provide strategic guidelines for designing virtual exhibitions that maximize user immersion and satisfaction, reaffirming the importance of storytelling content that emphasizes interest and emotion.

## 1. Introduction

The advancement of digital technology has introduced a new paradigm in exhibition culture, with virtual reality (VR) technology, in particular, enabling immersive exhibition experiences that transcend physical limitations. Amid these changes, digital storytelling has emerged as a key element—not only delivering information but also fostering emotional connection and continuous engagement from users.

Digital storytelling is a digital-based narrative delivery method that conveys narrative messages through various media such as text, images, video, sound, and interaction. It enables emotional immersion and interaction with users and facilitates the delivery of content with educational and cultural purposes [1,2,3].

Digital storytelling enhances users’ memory and comprehension by fostering emotional empathy, thereby providing a more powerful experience [1]. At the same time, it stimulates interest and encourages continued immersion by combining educational value with compelling storytelling [4]. For this to be effective, the structural design of the story is crucial. Ohler [3] emphasized that digital storytelling is not just about using technology, but also about designing a fundamental “story arc”—including introduction, conflict, climax, and resolution—as the core of narrative construction. Georgakopoulou [5] argued that traditional narrative structures must be reconstructed in digital environments and emphasized the need for plot design that reflects causal development and emotional curves, considering user participation. Furthermore, Murray [6] proposed that digital narratives possess four key characteristics: procedural, participatory, spatial, and encyclopedic design, highlighting the importance of structural planning that takes these into account. Researchers focusing on structural design have proposed key factors for effective narrative delivery and have empirically verified the narrative elements essential for digital storytelling in VR environments. These studies assert that such elements play a major role in shaping user experience.

In this context, Kim [7] found that interest-based elements increase user engagement and enhance immersion, while several scholars have argued that emotional elements activate users’ affective responses and play a significant role in increasing enjoyment and continuance intention [8,9,10]. Regarding educational value, studies have verified that educational elements improve users’ learning outcomes and sense of presence, ultimately enhancing their intention to revisit [11,12]. These findings demonstrate the complex influence of each digital storytelling component on user experience and reinforce the importance of digital storytelling in VR exhibitions.

In exhibitions utilizing VR, understanding the relationships among presence, immersion, enjoyment, and continuance intention is essential in comprehending the user experience. However, previous studies have mainly focused on isolated factors or have lacked a systematic analysis of the structural relationships among these factors.

First, there is insufficient analysis of the specific role of digital storytelling. While Liu and Sutunyarak [13] explored the impact of immersive technology in museums on visitors’ behavioral intentions, their study lacked an in-depth examination of the role of digital storytelling itself.

Second, many studies show a technological bias. For example, Wu et al. [14] investigated factors affecting the continuance intention of young users in a metaverse museum using digital twin technology. However, this research emphasized technological aspects rather than exploring the impact of digital storytelling.

Third, there is a lack of systematic structural analysis. For example, Jiang et al. [15] analyzed the factors influencing users’ continuous behavioral intentions in virtual cultural heritage tourism by integrating experience economy theory and the Stimulus-Organism-Response (SOR) model. Although they identified multiple influential factors, their study still lacked an integrated structural analysis of how digital storytelling specifically contributes to such intentions.

Fourth, there is limited consideration of continuance intention. For example, Xia [16] examined how online digital art exhibitions affect tourists’ on-site visit intentions. While the study revealed that online exhibitions influence tourist behavior, it did not analyze how digital storytelling contributes to users’ continued engagement.

The common limitation across these studies is that they fail to comprehensively analyze the structural relationships among key digital storytelling factors—such as presence, immersion, enjoyment, and continuance intention—in VR exhibition environments.

To fill this research gap, the present study aims to empirically examine how the key features of digital storytelling in VR environments affect presence, immersion, enjoyment, and continuance intention. By doing so, this research seeks to demonstrate the effectiveness of digital storytelling and contribute to both academic understanding and practical applications in the context of VR exhibitions.

The objectives of this study are as follows. First, to examine the effects of interest, emotion, and educational value in VR-based digital storytelling on presence and immersion. Second, to analyze how presence and immersion influence enjoyment and continued usage intention. Third, to empirically validate the impact of enjoyment on continued usage intention. Through these objectives, this study aims to demonstrate the effectiveness of digital storytelling in virtual exhibition environments and contribute to relevant academic fields and practical applications. In particular, by clearly defining the structural role of digital storytelling in VR exhibitions, this study aspires to provide both academic and practical value.

This study aims to achieve the following objectives:

1. To examine the effects of interest, emotion, and educational value in VR-based digital storytelling on presence and immersion.
2. To analyze the influence of presence and immersion on enjoyment and continuous usage intention.
3. To empirically validate the impact of enjoyment on continuous usage intention.

Through these objectives, this research seeks to demonstrate the effectiveness of digital storytelling in virtual exhibition environments and provide meaningful contributions to both theoretical and practical fields. Specifically, by clearly identifying the structural role of digital storytelling in VR exhibitions, this study offers both academic and industry-relevant insights.

## 2. Related Studies

### 2.1. The Concept and Role of Digital Storytelling in VR Exhibitions

Storytelling is one of the oldest forms of human communication, serving as a means to share knowledge, emotions, and cultural values [17]. Traditional storytelling follows a structured narrative format, often based on Aristotle’s three-act structure, which consists of a beginning, middle, and end, creating a coherent and engaging experience (Aristotle, 350 BCE). Additionally, Propp [18] analyzed folktales and identified common narrative functions, emphasizing the structural consistency of storytelling across cultures.

The definition of digital storytelling varies among scholars, as shown in Table 1. However, a common characteristic is that storytelling has evolved into digital storytelling with the advancement of digital technology, incorporating various digital media elements such as text, audio, video, and interactive components [1].

Digital storytelling enhances immersion and engagement, particularly in virtual reality (VR) environments, where users can interact with the narrative in a multi-sensory manner [25]. According to Murray [6], digital narratives in immersive environments enable greater interactivity, allowing users to influence the storyline and experience a heightened sense of presence. Furthermore, Lambert [1] emphasized that emotional engagement is a key component of digital storytelling, fostering deeper connections between the audience and the content.

VR (virtual reality) exhibitions have established themselves as a significant medium for providing highly immersive experiences for users. In particular, digital storytelling in VR environments plays a crucial role in eliciting users’ psychological responses, transforming passive spectators into active participants through immersion, interactivity, and emotional connection [6,25,26,27]. Existing studies have reported that digital storytelling serves as a vital mechanism for enhancing the quality of user experience beyond simple information delivery [28,29], a role that becomes even more pronounced in VR exhibitions.

Digital storytelling has been studied from various perspectives by different scholars. Researchers have identified key components of digital storytelling, including interest [22,30], emotion [31,32,33,34,35], educational engagement [31,36,37,38], interactivity [39,40,41,42], narrative structure [6,25,43,44], visual elements [45,46,47], and technological affordances [48,49,50,51].

This study aims to examine the key variables of digital storytelling—interest, emotion, and educational value—proposed in the study by Ding & Lee [31]. Among these key elements, interest plays a significant role in capturing users’ attention and enhancing their sense of presence. In VR environments, engaging storytelling elements serve as a decisive factor in reinforcing user immersion [52,53,54], which is directly linked to presence [55,56,57]. For instance, interactive narrative design encourages users to actively engage with VR content, thereby increasing their level of immersion [58]. This process allows users to transition from being mere information consumers to active participants in the narrative, ultimately contributing to sustained engagement and deeper immersion [25].

Emotional elements also play a crucial role in the VR environment. Emotional storytelling elements induce users’ emotional immersion, thereby enhancing the sense of presence [58,59,60,61]. VR content that includes emotional experiences tends to leave a stronger impression on users’ memories, ultimately strengthening memory retention and fostering a favorable attitude [62,63]. Additionally, it can serve as a factor that increases users’ intention to revisit or continue using the content in the future [63,64,65]. Moreover, VR experiences can positively influence users’ emotional responses and memory, with highly immersive VR content particularly enhancing emotional reactions and facilitating the internalization of information [66].

According to previous studies, educational elements have a significant impact on immersion, presence, and satisfaction; in particular, story-based learning enhances users’ emotional engagement, thereby facilitating better understanding and learning outcomes [67,68]. Specifically, digital storytelling imbued with historical and cultural narratives is reported to foster emotional empathy in visitors, thereby strengthening immersion and enabling educational messages to be retained for a longer period of time [69,70,71]. The interactivity of VR technology, by allowing visitors to directly experience and explore content, emerges as a key factor in boosting experiential satisfaction as well as intentions for revisits or continued use [71,72,73,74]. In particular, VR environments offering high levels of sensory and realistic elements are effective in heightening learners’ sense of presence, thereby maximizing learning outcomes [75].

A review of prior studies suggests that digital storytelling has emerged as a key factor influencing users’ immersion and satisfaction in VR exhibition environments. However, existing research has several limitations, including insufficient analysis of the specific role of digital storytelling, a tendency to focus on technological aspects, a lack of systematic analysis of structural relationships, and limited consideration of continuance intention. To address these research gaps, the primary objective of this study is to empirically examine how VR-based digital storytelling influences presence, immersion, enjoyment, and continued usage intention.

In particular, this study seeks to clarify the structural role of digital storytelling in VR exhibition settings. To that end, we analyze the effects of three core attributes—interest, emotion, and educational value—on presence, immersion, enjoyment, and continued usage intention. By doing so, this research aims to demonstrate the effectiveness of digital storytelling in VR exhibitions and contribute both to the relevant academic discourse and practical applications.

### 2.2. The Relationship Between Digital Storytelling, Presence, and Immersion in VR Exhibitions

VR technology is transforming user experiences in an innovative way, particularly by enhancing immersion when combined with digital storytelling in exhibition environments. In this context, digital storytelling is emerging as a key element that goes beyond mere information delivery to foster emotional connections and sustained user engagement. Lee [76] defined digital storytelling as “a short multimedia narrative that meaningfully integrates text, images, audio, and video, emphasizing the ability to interpret, utilize, and appreciate digital video”. Furthermore, Rhee et al. [77] highlighted that digital storytelling can enhance user immersion and satisfaction.

Interactive digital storytelling in VR increases user participation and elicits emotional responses, shaping a positive user experience [78]. Specifically, it enhances mediated presence in VR environments and improves users’ behavioral responses, making it particularly effective in educational and training scenarios [79,80].

Digital storytelling is a method of delivering narratives to users through digital media, playing a crucial role in enhancing interactivity within VR environments [78,81]. Interactive narratives encourage users to actively engage with VR content, thereby amplifying immersive experiences [82]. Furthermore, VR allows users to experience stories from another person’s perspective, increasing immersion. Presence, engagement, and user experience (UX) serve as key predictors of participation in VR storytelling environments [83,84]. These elements—immersion, presence, and UX—act as primary predictive factors that positively influence user engagement in VR storytelling settings [83]. Furthermore, engaging storytelling in VR environments serves as a critical factor in enhancing user immersion, which is directly linked to presence [85,86]. According to Oh & Kong [85], high-quality, narrative-driven content that fosters emotional attachment is essential for strengthening user immersion in VR environments. They reported that such content has a greater impact than merely possessing advanced VR technology, providing users with stronger emotional connections and an enhanced sense of presence.

Presence refers to the psychological perception of experiencing a virtual environment as if it were the real world, which is closely related to immersion [87]. In highly present VR environments, users exhibit greater task engagement [88], ultimately leading to increased enjoyment and improved learning outcomes [89]. Conversely, Makransky & Lilleholt [90] warned that, in VR-based learning environments, the excessive or unnatural presentation of educational materials could disrupt immersion and presence, potentially causing adverse effects on learners.

The relationship between presence and immersion has been a significant topic in VR research. Presence is the degree to which users perceive a virtual environment as real, and it is directly connected to immersion [87]. Higher levels of presence lead users to perceive the virtual environment more similarly to reality, thereby increasing their immersion [91]. According to Cummings & Bailenson [92], users in highly present VR environments interact more actively with their surroundings, and heightened vividness enhances their sense of presence. Similarly, Lombard & Ditton [35] argued that presence and immersion are closely linked to emotional responses (e.g., enjoyment and satisfaction), stating that higher immersion or presence leads to more favorable assessments of VR experiences.

These prior research findings clearly demonstrate the impact of digital storytelling components on presence and immersion, further validating the effectiveness of digital storytelling in VR exhibition environments. Based on these studies, this research systematically analyzes the complex influence of digital storytelling components (interest, emotion, and educational value) on presence and immersion, aiming to provide both academic and practical contributions.

### 2.3. The Relationship Between Presence, Immersion, Enjoyment, and Continued Usage Intention in VR Exhibitions

VR (virtual reality) technology has revolutionized user experiences, particularly in exhibition environments, where the interaction between presence, immersion, enjoyment, and continued usage intention plays a crucial role. In VR, presence enhances users’ ability to recall experiential content and positively influences their behavior [93,94].

Choi & Noh [95] suggested that presence strengthens the flow and arousal within VR environments, leading to increased enjoyment and a higher intention to continue using the platform. Similarly, Weibel & Wissmath [96] explored the role of spatial presence and flow as key factors of immersion, emphasizing that spatial presence is a critical component of immersion, which in turn plays a significant role in enhancing enjoyment.

Immersion also influences enjoyment and continued usage intention. Csikszentmihalyi [97] defined flow as “a state in which users become fully absorbed in an activity, losing awareness of external stimuli”, arguing that this state enhances user enjoyment and satisfaction. Kowalczuk et al. [98] demonstrated that immersion and enjoyment lead to higher reuse and purchase intentions, highlighting the importance of interactive and immersive experiences. Canio et al. [99] further emphasized that immersion and presence in VR tours directly impact enjoyment, which subsequently influences user satisfaction and visit intentions. Similarly, Anaya-Sánchez et al. [100] found that immersion plays a crucial role in improving user experience and satisfaction, thereby positively affecting visit intentions.

Immersion in VR is a critical factor in enhancing user satisfaction. Studies indicate that when users experience a genuine sense of embodiment and novelty in VR, their perceived usefulness and satisfaction increase, leading to a stronger intention to continue using VR [101,102].

Enjoyment also directly impacts continuous use intention. Lin et al. [103] reported that enjoyment enhances user satisfaction, which ultimately leads to continued usage. İlkan et al. [104] stated that enjoyment positively influences user satisfaction and the sustained use of applications. Binowo et al. [105] confirmed that, in virtual exhibitions, enjoyment significantly affects user satisfaction and continuous use intention. Lee et al. [106] found that the strength of social interaction and social bonding enhances perceived enjoyment, which in turn has a positive impact on the continued use of VR devices. Various studies have demonstrated that enjoyment is a key antecedent influencing user adoption and continued usage intention [107,108].

Synthesizing these prior studies, presence has been identified as a key factor that strengthens continued usage intention through immersion and enjoyment. However, existing research has often focused on specific variables or failed to systematically analyze structural relationships. To address this research gap, this study aims to empirically examine the relationships among presence, immersion, enjoyment, and continued usage intention, providing deeper insights into their interconnections within VR exhibition environments.

## 3. Research Model and Hypothesis Setting

To validate the conceptual framework of this study, a research model was developed based on a comprehensive review of relevant prior studies. Key measurement variables were identified, and their interrelationships were established in accordance with theoretical foundations and empirical findings. Specifically, the digital storytelling elements in the virtual exhibition were categorized into three main factors: interest, emotion, and educational value. These components were selected based on their frequent appearance and validated impact in previous research on immersive media and user engagement.

**Figure 1:**
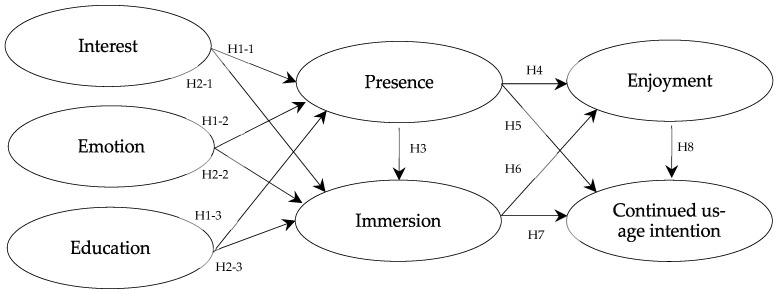
Research model to verify the relationship between digital storytelling factors and presence, immersion, enjoyment, and intention to continued usage intention.

Furthermore, to examine how these storytelling elements influence users’ psychological and behavioral responses, the model incorporated four outcome variables: presence, immersion, enjoyment, and continued usage intention. The structural relationships among these variables were formulated to assess the dynamics of user experience within VR-based exhibitions. The final research model was designed as illustrated in Figure 1.

Based on the aforementioned prior studies, the following research hypotheses have been established. H1: VR digital storytelling elements (interest, emotion, and educational value) will have a positive (+) effect on presence. H1-1: Interest will have a positive (+) effect on presence. H1-2: Emotion will have a positive (+) effect on presence. H1-3: Education will have a positive (+) effect on presence. H2: VR digital storytelling elements (interest, emotion, and educational value) will have a positive (+) effect on immersion. H2-1: Interest will have a positive (+) effect on immersion. H2-2: Emotion will have a positive (+) effect on immersion. H2-3: Education will have a positive (+) effect on immersion. H3: Presence will have a positive (+) effect on immersion. H4: Presence will have a positive (+) effect on enjoyment. H5: Presence will have a positive (+) effect on continued usage intention. H6: Immersion will have a positive (+) effect on enjoyment. H7: Immersion will have a positive (+) effect on continued usage intention. H8: Enjoyment will have a positive (+) effect on continued usage intention.

## 4. Method

### 4.1. Research Subjects and Data Collection

For this study, the online VR version of the special exhibition “Pinnacle of Property: The Uigwe, Records of the State Rites of the Joseon Dynasty” produced by the National Museum of Korea was selected. The reasons for this selection were twofold: first, the exhibition had already been validated through its offline version, which attracted significant public interest, with a total of 84,283 visitors between 1 November 2022 and 29 January 2023, averaging approximately 926 visitors per day [109]; and, second, the credibility of the exhibition, being produced by the National Museum of Korea, ensured the reliability of its VR content.

**Figure 2:**
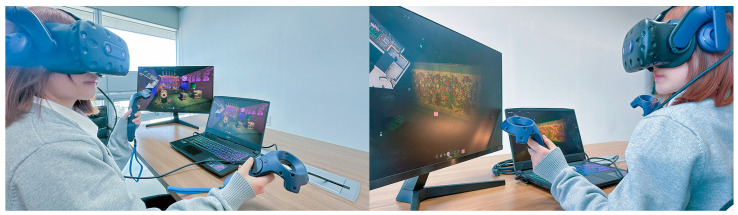
Experiential scene of a VR experiment using the metaverse.

To examine how digital storytelling operates through VR exhibition content, a survey was conducted from 15 June to 27 July 2024, targeting 179 university students enrolled at G University in Gyeonggi Province, South Korea. Before the experiment, participants were informed about the research objectives, experiment procedures, and precautions, and only those who agreed to the consent form proceeded with the experiment. Before the survey, participants received instructions on how to operate the device and experience the content. They then wore VR headsets (HTC VIVE Pro-2), as shown in Figure 2, and spent about 5 min familiarizing themselves with the controls.

Given the nature of VR content, cyber sickness was a potential issue. Therefore, participants were informed in advance that they could withdraw from the experiment at any time if they experienced physical fatigue, dizziness, or nausea during the experiment. This study was approved by the Institutional Review Board (IRB) of C University (Approval No. 1044396-202303-HR-043-01). Once the participants were fully prepared, they explored the VR exhibition (https://www.museum.go.kr/museum/2023/uigwe_virtualtour/, accessed on 6 April 2024) for an adequate amount of time. Afterward, the survey objectives and instructions were explained, and the survey was distributed.

For data collection, a self-administered survey method was employed, where participants directly filled out the questionnaire. Among the 179 respondents, 6 responses were excluded due to invalid or insincere answers (e.g., missing values, incomplete responses), resulting in a final dataset of 173 valid responses for analysis.

#### Analysis Results

The demographic analysis of the survey respondents revealed that female participants (72.3%) outnumbered male participants (27.7%), with an average age of 21.8 years. In terms of VR usage experience over the past year, 68.2% of respondents had used VR between 1 and 5 times, making this the most common frequency. Regarding monthly VR usage, 91.1% of participants reported using VR less than twice per month, followed by 3–4 times per month (3.9%). When asked about their primary purpose for using VR, gaming (74.3%) was the most common response, followed by education and learning (10.8%).

### 4.2. Operational Definitions and Measurement Tools of Variables

#### 4.2.1. Digital Storytelling Factors

In this study, the measurement items for VR digital content factors were refined and adapted based on the research of YI & Li [31] to align with the study’s objectives. To evaluate these factors, a 5-point Likert scale was employed, where participants rated their responses from 1 (Strongly Disagree) to 5 (Strongly Agree).

The study utilized a total of 11 measurement items, categorized into three key dimensions: interest, emotion, and educational value. The interest dimension was assessed through four items, including statements such as “This exhibition stimulates my interest” and “I felt a sense of anticipation for new discoveries while viewing the exhibition”. These items aimed to capture the extent to which the exhibition engaged and intrigued participants.

Similarly, the emotion dimension was measured using four items designed to evaluate the participants’ emotional engagement. Examples of these statements include “I was emotionally moved by the museum exhibition” and “I felt a strong sense of empathy with the exhibition content”. These items aimed to assess the emotional impact of the VR exhibition on visitors.

The educational value dimension was assessed using three items, focusing on the perceived learning outcomes of the exhibition. An example statement from this category is “The museum exhibition enriched my knowledge”. These items sought to determine the extent to which participants felt they gained valuable information from the exhibition.

By structuring the measurement items across these three dimensions, the study effectively captured the role of VR digital storytelling in shaping user experience within virtual exhibitions.

#### 4.2.2. Presence

In this study, presence was measured by modifying and refining the spatial presence factor from the scale developed by Lessiter et al. [110] and later applied in Kang [111]. These modifications were made to better align with the context of this research.

The measurement consisted of four items, designed to assess the sense of physical spatial presence experienced by participants in the VR exhibition. Example statements included “I felt as if I were interacting with the exhibition pieces” and “The exhibits felt so close that I could reach out and touch them”. To evaluate responses, a 5-point Likert scale was used, where 1 indicated ‘Strongly Disagree’ and 5 indicated ‘Strongly Agree’.

By incorporating these refined items, this study aimed to capture how effectively VR exhibitions create a sense of spatial presence and realism for users.

#### 4.2.3. Immersion

In this study, measurement items were adapted from the works of Hudson et al. [101], Jennett et al. [112], and Yoon et al. [113] to better fit the research context. These modifications aimed to more accurately assess the immersion experience in a VR exhibition setting.

The final measurement consisted of three items, designed to evaluate the extent to which participants felt deeply engaged and disconnected from their external surroundings while experiencing the VR exhibition. Example statements included “I felt disconnected from the outside world while experiencing the VR exhibition” and “I felt completely immersed in the VR exhibition”.

Each item was assessed using a 5-point Likert scale, where 1 indicated “Strongly Disagree” and 5 indicated “Strongly Agree”. Through these measurements, the study sought to capture the depth of user immersion and engagement within the VR exhibition environment.

#### 4.2.4. Enjoyment

In In this study, measurement items were adapted from Venkatesh [114], Woo & Hwang [115], and Kim [116] to better align with the research objectives. These modifications were made to assess the level of enjoyment experienced during the VR exhibition.

The final measurement consisted of three items, designed to evaluate how enjoyable and engaging participants found the VR experience. Example statements included “Experiencing virtual reality is enjoyable” and “The VR experience is interesting”.

Each item was assessed using a 5-point Likert scale, where 1 indicated “Strongly Disagree” and 5 indicated “Strongly Agree”. Through these measurements, the study aimed to capture users’ enjoyment levels and their engagement with the VR exhibition.

#### 4.2.5. Continued Usage Intention

In this study, measurement items were adapted from Choi et al. [117] and Kim & Oh [118] to better align with the research objectives. These modifications were made to assess users’ continued usage intention toward VR technology.

The final measurement consisted of four items, designed to evaluate participants’ willingness to continue using VR devices in the future. Example statements included “I would like to use VR devices in the future” and “I am willing to actively use VR devices moving forward”.

Each item was assessed using a 5-point Likert scale, where 1 indicated “Strongly Disagree” and 5 indicated “Strongly Agree”. Through these measurements, the study aimed to capture users’ future intentions and willingness to engage with VR technology beyond the experimental setting.

### 4.3. Validation of Measurement Variables and Reliability

A confirmatory factor analysis was conducted to verify the validity of the variables and measurement items in this study.

To analyze the validity of the measurement variables, convergent validity and discriminant validity were examined.

First, convergent validity was verified by assessing the Average Variance Extracted (AVE) and Composite Reliability (CR) values. As shown in Table 2, the AVE values ranged from 0.808 to 0.869, meeting the recommended threshold of ≥0.5, while the CR values ranged from 0.927 to 0.952, exceeding the acceptable criterion of ≥0.7. These results confirm that convergent validity was established.

Next, discriminant validity was assessed using the AVE values and the squared correlations between variables. According to the results presented in Table 3, the highest correlation coefficient was found between “interest” and “continued usage intention”, with a correlation value of 0.834. The lowest square root of AVE was 0.900, which exceeded the highest correlation coefficient. Therefore, discriminant validity was confirmed, and hypothesis testing was conducted using the structural model.

## 5. Research Result

The results of the structural model analysis for hypothesis testing were as follows.

First, the model fit indices were evaluated, yielding the following results: χ^2^ = 424.773 (df = 260), *p* = 0.000 (*p* ≥ 0.05 is desirable), GFI = 0.842 (≥0.80 is acceptable), AGFI = 0.802 (≥0.80 is acceptable), RMR = 0.023 (<0.05 is acceptable), TLI = 0.942 (≥0.90 is acceptable), CFI = 0.950 (≥0.90 is acceptable), and RMSEA = 0.061 (≤0.10 is acceptable). Since all fit indices met or exceeded the recommended thresholds, the model was evaluated as a good fit.

**Figure 3:**
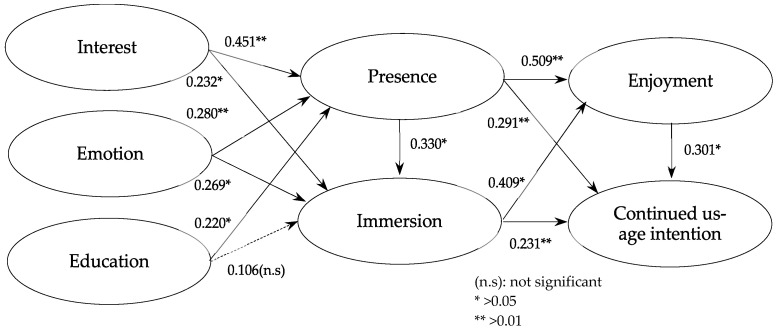
Result of path estimation.

The results of the hypothesis testing based on the research model are presented in Table 4 and Figure 3.

The analysis results indicate that the hypothesis “Digital storytelling using VR (interest, emotion, and educational value) will have a positive (+) effect on presence” was fully supported. Specifically, interest (β = 0.451, t = 4.805, *p* < 0.000), emotion (β = 0.280, t = 3.629, *p* < 0.000), and education (β = 0.220, t = 2.894, *p* < 0.004) had a significant positive impact on presence (H1-1, H1-2, H1-3).

Next, the hypothesis “Digital storytelling using VR (interest, emotion, and educational value) will have a positive (+) effect on immersion” (H2) was partially supported, as educational value did not show a statistically significant effect. The results show that interest (β = 0.232, t = 1.966, *p* < 0.05) and emotion (β = 0.269, t = 1.998, *p* < 0.05) had a significant positive impact on immersion (H2-1, H2-2), while education did not (H2-3). Furthermore, the hypothesis “Presence will have a positive (+) effect on immersion” (H3) was supported (β = 0.330, t = 2.417, *p* < 0.05). Additionally, the hypotheses “Presence will have a positive (+) effect on enjoyment” (H4) (β = 0.509, t = 4.424, *p* < 0.000) and “Presence will have a positive (+) effect on continued usage intention“ (H5) (β = 0.291, t = 3.744, *p* < 0.000) were both supported. Next, the hypotheses “Immersion will have a positive (+) effect on enjoyment” (H6) (β = 0.409, t = 2.643, *p* < 0.05) and “Immersion will have a positive (+) effect on continued usage intention“ (H7) (β = 0.231, t = 2.462, *p* < 0.05) were also supported.

Finally, the hypothesis “Enjoyment will have a positive (+) effect on continued usage intention” (H8) was supported (β = 0.301, t = 2.859, *p* < 0.001).

## 6. Results and Discussion

This study explored how interest, emotion, and educational value in digital storytelling influence presence, immersion, enjoyment, and continued usage intention in VR-based virtual exhibitions, using path analysis. The results show that most of the proposed hypotheses were confirmed, in line with previous studies.

### 6.1. Effects of Digital Storytelling on Presence

The hypothesis “Digital storytelling components (interest, emotion, and educational value) positively influence presence” was fully supported. Interest (β = 0.451, *p* < 0.000), emotion (β = 0.280, *p* < 0.000), and educational value (β = 0.220, *p* < 0.004) were all found to have significant positive effects on presence. These results are consistent with prior research, which suggests that engaging narratives capture and sustain audience attention, thereby increasing presence [119,120]. Additionally, emotional elements in storytelling enhance presence by eliciting affective responses from users [120,121], while educational storytelling provides new information that strengthens presence and encourages cognitive engagement [122,123]. In storytelling, interest, emotion, and education are essential for enhancing user immersion and presence [124,125,126]. When effectively incorporated, these elements enable users to deeply engage with the narrative and experience it as if it were real; therefore, content creators should integrate these components into digital storytelling to optimize user experiences [124,127].

### 6.2. Effects of Digital Storytelling on Immersion

The hypothesis “Digital storytelling components (interest, emotion, and educational value) positively influence immersion” was partially supported, as educational value did not show a significant effect on immersion. Interest (β = 0.232, *p* < 0.05) and emotion (β = 0.269, *p* < 0.05) had positive effects, aligning with findings that engaging storytelling fosters deeper user involvement and heightens immersion intensity [128]. Emotional engagement enables users to resonate with the story’s affective flow, deepening their immersion [84,129]. While educational storytelling is generally expected to help users connect with narratives by facilitating learning [130,131], this study found that education did not directly impact immersion. The non-significant impact of educational value on immersion may be interpreted through the lens of cognitive–sensory distinction and self-determination theory. Educational content, when heavily focused on achieving learning outcomes, may increase users’ cognitive load and reduce their sensory engagement. According to Deci and Ryan’s Self-Determination Theory, externally imposed educational goals can undermine users’ sense of autonomy, thereby decreasing intrinsic motivation and spontaneous exploratory behaviors. This may inhibit the affective and sensory immersion typically associated with VR experiences [132]. Consequently, educational storytelling that lacks emotional or narrative engagement may fail to provide the immersive richness required in virtual exhibition contexts. These findings suggest that designing digital storytelling with intrinsic motivation and autonomy-supportive features can enhance immersion.

### 6.3. Effects of Presence on Immersion, Enjoyment, and Continued Usage Intention

The hypothesis “Presence positively influences immersion” (H3) was supported (β = 0.330, *p* < 0.05), in line with prior studies [133,134]. Chang & Suh [135] found that higher presence enhances user immersion, while Barbieri et al. [133] reported that story-driven VR exhibitions strengthen presence, promoting early user engagement and positive learning experiences. These findings indicate that presence plays a crucial role in fostering immersion. Similarly, the hypotheses “Presence positively influences enjoyment” (H4) (β = 0.509, *p* < 0.000) and “Presence positively influences continued usage intention” (H5) (β = 0.291, *p* < 0.000) were also supported. Jang & Park [134] found that presence enhances enjoyment, which in turn strengthens continued usage intention, while Choi [136] emphasized the critical role of presence in fostering user satisfaction and sustained engagement. These results align with previous studies, confirming the positive influence of presence on enjoyment and continued usage intention.

### 6.4. Effects of Immersion and Enjoyment on Continued Usage Intention

The hypotheses “Immersion positively influences enjoyment” (H6) (β = 0.409, *p* < 0.05), “Immersion positively influences continued usage intention” (H7) (β = 0.231, *p* < 0.05), and “Enjoyment positively influences continued usage intention” (H8) (β = 0.201, *p* < 0.001) were all supported, consistent with prior research [32,137,138]. Kosa et al. [139] found that immersion satisfies users’ need for autonomy and competence, enhancing enjoyment, which in turn positively affects continued usage intention. Similarly, Csikszentmihalyi [97] argued that immersion maximizes enjoyment and personal fulfillment, while Hoffman & Novak [32] demonstrated that immersion enhances enjoyment, satisfaction, and long-term engagement.

Moreover, enjoyment is directly influenced by immersion and presence, making it a key determinant of sustained usage behavior [106,140]. Research suggests that social interaction and the intensity of social connectedness in VR environments enhance enjoyment, which subsequently influences continued usage intention. These findings reinforce the significant impact of immersion and enjoyment on sustained engagement in VR environments, highlighting the crucial role of affective experiences in shaping user behavior.

## 7. Conclusions

This study systematically analyzed the impact of the key elements of digital storytelling (interest, emotion, and education) on presence, immersion, enjoyment, and continued usage intention in VR-based virtual exhibitions. The findings empirically confirm that digital storytelling components have a complex influence on user experiences, with interest and emotion being the most significant factors in enhancing presence and immersion. Additionally, the study demonstrates that presence positively affects immersion, enjoyment, and continued usage intention, while immersion and enjoyment significantly contribute to sustained user engagement.

The finding that interest has the strongest influence on presence suggests that capturing users’ attention is a fundamental aspect of VR exhibition design. This underscores the importance of enhancing interactive storytelling elements and engaging narrative structures. To maintain user curiosity and encourage active exploration, nonlinear storytelling techniques, personalized exhibition pathways, and real-time feedback interactions should be integrated.

Moreover, the role of emotion in increasing presence highlights the importance of emotional engagement in strengthening users’ spatial perception within VR environments. This finding emphasizes that VR exhibitions should go beyond mere information delivery and focus on evoking emotional responses through immersive content. Therefore, high-resolution graphics, multidimensional 3D audio, haptic feedback, and narrative-driven interactivity should be strategically implemented to enhance emotional immersion.

Furthermore, the positive impact of presence on immersion, enjoyment, and continued usage intention indicates that immersive environmental design in VR exhibitions plays a critical role in shaping user experiences and revisit intentions. To maximize presence, VR exhibition designers should employ immersive spatial environments (e.g., 6DOF-based navigation and natural environmental responses), sensory feedback mechanisms (e.g., haptic technology and motion capture), and personalized storytelling experiences to create a multi-sensory engagement approach.

The significant influence of enjoyment on continued usage intention highlights the necessity of designing emotionally engaging VR exhibitions. Enhancing visual immersion through high-quality graphics, incorporating emotionally compelling narratives, and integrating gamification elements can contribute to long-term user engagement. This suggests that VR exhibitions should evolve from passive viewing spaces into immersive environments that maximize sensory and emotional experiences, ultimately fostering sustained user participation and higher revisit rates.

An intriguing finding of this study lies in its contrast with most prior research, which generally reported that the educational value of digital storytelling effectively enhances user immersion. Contrary to these previous findings, the present study suggests that the impact of educational content on immersion may be limited in certain contexts. The finding that educational value did not significantly influence immersion highlights an important limitation of information-centered content in VR exhibitions: its reduced capacity to foster sensory and emotional immersion. This result reflects a critical distinction between cognitive and sensory immersion. As users are exposed to larger amounts of information, they are more likely to prioritize achieving learning objectives over engaging in exploratory or emotionally resonant experiences, thereby diminishing their overall immersion.

To address these limitations, VR exhibitions should undergo a fundamental transformation in content and interface design. Specifically, adopting nonlinear, emotionally driven storytelling structures that empower users to influence the narrative can strengthen emotional engagement and immersion. Furthermore, integrating intrinsically motivated gamification strategies, such as mission-based learning and achievement systems, can make educational experiences more explorative and emotionally rewarding.

Prioritizing autonomy-supportive interfaces—offering self-directed exploration and adaptive narrative scaffolding—can further enhance intrinsic motivation and user engagement. Educational content should also be embedded within emotionally resonant, character-driven narratives, enabling users to learn through emotional empathy and situational experience rather than through passive information absorption.

Thus, to fully leverage the educational potential of VR exhibitions, it is essential to shift from traditional information transmission models to more immersive, story-based, gamified, and experiential approaches. This balanced strategy can simultaneously optimize both user immersion and learning outcomes.

Overall, these findings indicate that future VR exhibition design must transcend simple information delivery, evolving into integrated immersive environments where sensory engagement and educational experience are harmoniously fused. This perspective offers critical implications for the development of next-generation digital storytelling-based educational programs.

This study further confirms that presence significantly influences immersion, enjoyment, and continued usage intention, reinforcing the crucial role of environmental and perceptual factors in shaping user behavior and satisfaction in VR exhibitions. High levels of presence enhance spatial perception and sensory engagement, allowing users to experience more realistic and emotionally immersive exhibitions. In particular, the sophistication of visual and auditory elements, along with spatial interaction mechanisms, are key contributors to heightened presence, which, in turn, strengthens users’ emotional responses and overall engagement.

Moreover, the study suggests that VR exhibition design should prioritize immersive spatial interactions and refine sensory elements to maximize presence. The positive relationship between presence and continued usage intention indicates that advancements in VR exhibition technology and personalized content development can significantly enhance long-term user retention. Future VR exhibitions should adopt dynamic exhibition designs based on user behavior patterns and sensory responses, integrating interactive narratives and feedback-driven mechanisms to enhance immersion. These findings emphasize that VR exhibitions should evolve beyond static information displays and transform into experience-driven environments that evoke deep emotional and psychological engagement.

The findings also confirm that immersion significantly affects enjoyment and continued usage intention, suggesting that deeper engagement leads to stronger emotional responses and sustained interest in VR exhibitions. This highlights the need for VR exhibition designs that maintain user immersion over extended periods, incorporating story-driven exhibition layouts, highly interactive interfaces, and self-guided exploration options that encourage active participation.

Additionally, the study confirms that enjoyment plays a crucial role in influencing continued usage intention, emphasizing the need to maximize emotional engagement within VR exhibitions. To achieve this, VR exhibitions should integrate multi-sensory media, emotion-driven storytelling techniques, and naturalistic exploration experiences to enhance user satisfaction. Moreover, balancing emotional design and interactivity is essential in ensuring that VR exhibitions encourage active exploration and participation rather than passive viewing.

### 7.1. Academic and Practical Contributions

This study emphasizes the critical role of digital storytelling in enhancing user experience and strengthening continuous usage intention in VR exhibitions. By systematically analyzing the structural effects of key digital storytelling elements—interest, emotion, and education—on presence, immersion, enjoyment, and continuance intention, this research fills a gap in VR exhibition studies by providing a more comprehensive analysis.

Academically, this study makes a theoretical contribution by clarifying the structural relationships between digital storytelling components and user experience, thereby advancing research in related fields. In particular, it systematically examines how interest, emotion, and education influence presence, immersion, enjoyment, and continuance intention, providing empirical evidence for the effectiveness of digital storytelling in VR exhibitions.

From a practical perspective, this study offers strategic insights for maximizing user immersion and satisfaction in virtual exhibition design. It reaffirms the importance of developing storytelling content that prioritizes interest and emotion, ensuring that VR exhibitions deliver more engaging and immersive experiences. By applying these insights, industry professionals can create more effective digital exhibition environments that foster sustained visitor engagement and long-term participation.

### 7.2. Limitations and Future Research

Despite its several academic contributions, this study has some limitations.

First, the sample was limited to university students in their early twenties from a specific region, which may restrict the generalizability of the findings. Future studies should include participants from diverse age groups, geographic locations, and digital literacy backgrounds to enhance the robustness of the results.

Second, although the education did not show a significant effect on immersion in this study, this relationship may vary depending on sample characteristics and experimental conditions. It may also be influenced by factors such as the structure of the content and the amount of information provided. Therefore, further research is needed to investigate how different educational content delivery methods impact user immersion in VR exhibitions.

Additionally, this study employed VR content developed by the National Museum of Korea, which ensured content reliability and neutrality. However, as contemporary VR exhibition content increasingly integrates advanced UI/UX design, production techniques, and cutting-edge technology, the content used in this study may not fully represent the current state of VR exhibition environments. Future studies should comprehensively analyze a broader range of VR exhibition content to more precisely identify the role of storytelling in VR-based cultural experiences.

Finally, since the VR content used in this study does not fully reflect the diversity of existing VR exhibitions, future research should compare and analyze how various VR platforms and content types affect user experience and immersion. Moreover, as VR technology continues to evolve, further studies are needed to explore how emerging digital storytelling elements influence user experiences. Such research will contribute to the advancement of immersive cultural experiences in virtual environments.

## Figures and Tables

**Table 1:** Definition of digital storytelling.

Scholars	Definitions	Key Features
Lambert [1]	The process of expressing personal stories visually and audibly through digital technology.	Personal Experience-Centered, Utilization of Multimedia, Inducing Emotional Empathy
Ohler [3]	Digital storytelling is the process of creating and delivering stories using digital tools, which can also be utilized for educational purposes.	Emphasis on Creativity, Educational Applications, Collaborative Storytelling
Couldry [19]	Digital storytelling is the process of reconstructing and sharing human experiences through digital technology.	Reconstruction of Experience, Technological Support, Shareability
Robin [2]	Digital storytelling is an activity where users share and disseminate their stories on online platforms.	Utilization as a Learning Tool, Multimodal Expression, Public Accessibility
Burgess [20]	Digital storytelling is an activity where users share and disseminate their stories on online platforms.	Use of Social Media, User-Generated Content, Network Effect
Alexander [21]	Digital storytelling integrates various media elements to create an interactive narrative environment.	Combination of Technology and Human Experience, Expression in Various Formats, Potential for Dissemination
Sadik [22]	Digital storytelling is an educational tool designed to develop learners’ creativity and critical thinking.	Creativity Development, Motivation for Learning, Collaborative Learning
Frazel [23]	Digital storytelling is a narrative format that combines multimedia elements to enhance students’ learning experiences.	Multimedia Expression, Emphasis on Audiovisual Elements, Emotional Connection
Lundby [24]	Digital storytelling is a narrative practice that conveys social meaning through the use of media technology.	Social Message Delivery, Utilization of Media Technology, Community Building

**Table 2:** The result of confirmatory factor analysis.

Paths			Estimate	S.E.	C.R.	AVE
INT1 *	<-	Interest	0.799	0.171	0.947	0.818
INT2	<-		0.841	0.117		
INT3	<-		0.825	0.132		
INT4	<-		0.829	0.138		
EMO1 *	<-	Emotion	0.846	0.124	0.944	0.808
EMO2	<-		0.878	0.096		
EMO3	<-		0.790	0.155		
EMO4	<-		0.751	0.193		
EDU1	<-	Education	0.841	0.140	0.952	0.869
EDU2	<-		0.929	0.067		
EDU3	<-		0.851	0.140		
PRE1 *	<-	Presence	0.843	0.126	0.950	0.827
PRE2	<-		0.820	0.133		
PRE3	<-		0.785	0.177		
PRE4	<-		0.862	0.104		
IMM1 *	<-	Immersion	0.794	0.183	0.942	0.844
IMM2	<-		0.865	0.118		
IMM3	<-		0.884	0.099		
ENJ1 *	<-	Enjoyment	0.830	0.125	0.927	0.809
ENJ2	<-		0.760	0.200		
ENJ3	<-		0.816	0.130		
CON1 *	<-	Continued usage intention	0.838	0.120	0.949	0.823
CON2	<-		0.805	0.155		
CON3	<-		0.815	0.143		
CON4	<-		0.815	0.158		

Model fit: χ^2^ = 351.486 (df = 250, *p* < 0.00), GFI = 0.863, AGFI = 0.821, RMR = 0.016, TLI = 0.963, CFI = 0.969, RMSEA = 0.049. * INT: interest; EMO: emotion; EDU: education; PRE: presence; IMM: immersion; ENJ: enjoyment; CON: continued usage intention.

**Table 3:** Results of correlation analysis.

	INT	EMO	EDU	PRE	IMM	ENJ	CON
INT	0.911						
EMO	0.663	0.908					
EDU	0.698	0.601	0.932				
PRE	0.775	0.706	0.680	0.914			
IMM	0.728	0.709	0.639	0.781	0.919		
ENJ	0.721	0.680	0.735	0.750	0.716	0.900	
CON	0.834	0.723	0.790	0.811	0.775	0.809	0.907

* The values on the diagonal represent the square root of the AVE (Average Variance Extracted) for each variable. * The values below the diagonal represent the correlation coefficients. * INT: interest; EMO: emotion; EDU: education; PRE: presence; IMM: immersion; ENJ: enjoyment; CON: continued usage intention.

**Table 4:** Results of the structural equation model.

Hypotheses		Paths			Estimate	S.E.	C.R.	*p*-Value	Supported
**H1**	H1-1	Interest	->	Presence	0.451	0.094	4.805	0.000	**Yes**
	H1-2	Emotion	->		0.280	0.077	3.629	0.000	**Yes**
	H1-3	Education	->		0.220	0.076	2.894	0.004	**Yes**
**H2**	H2-1	Interest	->	Immersion	0.232	0.118	1.966	0.049	**Yes**
	H2-2	Emotion	->		0.269	0.093	2.907	0.004	**Yes**
	H2-3	Education	->		0.106	0.087	1.221	0.222	**No**
**H3**		Presence	->	Immersion	0.330	0.136	2.417	0.016	**Yes**
**H4**		Presence	->	Enjoyment	0.509	0.115	4.424	0.000	**Yes**
**H5**		Presence	->	Continued usage intention	0.291	0.109	3.744	0.000	**Yes**
**H6**		Immersion	->	Enjoyment	0.409	0.110	2.643	0.008	**Yes**
**H7**		Immersion	->	Continued usage intention	0.231	0.094	2.462	0.014	**Yes**
**H8**		Enjoyment	->	Continued usage intention	0.301	0.105	2.859	0.004	**Yes**

Model fit: χ^2^ = 424.773 (df = 260, *p* < 0.000), GFI = 0.842, AGFI = 0.802, RMR = 0.023, TLI = 0.942, CFI = 0.950, RMSEA = 0.061.

## Data Availability

Data are available on request from the authors.
